# Skewed Brownian Fluctuations in Single-Molecule Magnetic Tweezers

**DOI:** 10.1371/journal.pone.0108271

**Published:** 2014-09-29

**Authors:** Daniel R. Burnham, Iwijn De Vlaminck, Thomas Henighan, Cees Dekker

**Affiliations:** Delft University of Technology, Kavli Institute of Nanoscience, Department of Bionanoscience, Delft, The Netherlands; Swiss Federal Institute of Technology Zurich, Switzerland

## Abstract

Measurements in magnetic tweezers rely upon precise determination of the position of a magnetic microsphere. Fluctuations in the position due to Brownian motion allows calculation of the applied force, enabling deduction of the force-extension response function for a single DNA molecule that is attached to the microsphere. The standard approach relies upon using the mean of position fluctuations, which is valid when the microsphere axial position fluctuations obey a normal distribution. However, here we demonstrate that nearby surfaces and the non-linear elasticity of DNA can skew the distribution. Through experiment and simulations, we show that such a skewing leads to inaccurate position measurements which significantly affect the extracted DNA extension and mechanical properties, leading to up to two-fold errors in measured DNA persistence length. We develop a simple, robust and easily implemented method to correct for such mismeasurements.

## Introduction

Magnetic tweezers tether a single macromolecule between a surface and a superparamagnetic microsphere in order to apply piconewton forces and detect positional changes. Such positional changes can inform researchers about the mechanical properties of the macromolecule or its interaction with small molecules. Since its invention [1], the technique has been extensively employed to address biophysical problems [2] and developments to extend the instrument capabilities continue. Measurements of microsphere position are possible to a precision of 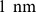 [3], at high sampling speeds of 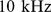 [4], and with multiplexing abilities [5]. Unique to magnetic tweezers is the inherent ability to fix angular position and hence apply torque to molecules. Furthermore, measurements are not merely limited to position but can include the ability to measure angular position, enabling torque measurement [6].

Stretching single molecules, typically DNA, is standard practice in magnetic tweezers instruments. The microsphere position is used to measure DNA end-to-end length and thus deduce any interactions that may be occurring. For example, plectonemes are inferred from the decrease in DNA end-to-end length as a function of supercoiling density, or polymerase activity is deduced from observations of an increasing end-to-end length as double-stranded DNA is converted to single-stranded DNA [7].

Furthermore, position fluctuation measurements can be used to deduce the mechanical properties of the tether by studying the force-extension behaviour; i.e. the mechanical extension of DNA at a given applied force. This behaviour is well described by a worm-like chain (WLC) entropic spring [8], [9] and is characterised by the contour length, 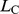, and persistence length, 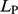. These mechanical properties have been described in detail, including the dependence on temperature, pH, and monovalent and multivalent salt [10]–[12]. The 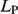 and 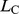 values first provide a confidence check that the molecule under study observes the expected behaviour, i.e. that the tether is a single DNA molecule of correct length. Secondly, the 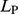 and 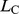 describe the stiffness and length of DNA, respectively, which have clear physiological consequences in many important biological processes such as nucleosome wrapping [13], protein-DNA binding interactions [14], and topological structure and dynamics [15].

As mentioned above, all measurements of end-to-end length and mechanical properties are dependent upon the basic readout mechanism for magnetic tweezers; an accurate measurement of microsphere position through time. If a bias occurs here, the DNA end-to-end length, and consequently the deduced interactions and mechanical properties will be misinterpreted.

This study reports a common bias of this type. Incorrectly, in standard analysis one assumes that the central limit theorem holds true and the arithmetic mean of axial position fluctuations represents the position of the microsphere. However, we show that one must take into account external interaction potentials, such as DNA elasticity and hydrodynamic coupling near surfaces. These interactions create biases that result in skewness in the axial position fluctuations of the microsphere. We provide simple improvements to the standard analysis that the experimenter can implement in order to correct the biases. Our analysis is supported by evidence from both experiments and numerical simulations. If overlooked, the bias can cause severe mismeasurements in the axial position of tethered microspheres in magnetic tweezers experiments and lead to significant errors. While the mistake is subtle, the precision typical of contemporary apparatus can reveal significant inaccuracies and misinterpretations, for example, up to a factor of two error in 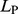.

## Materials and Methods

A detailed description of the constructs and tethering methods are given in file S1.

### Magnetic tweezers

**Figure 1:**
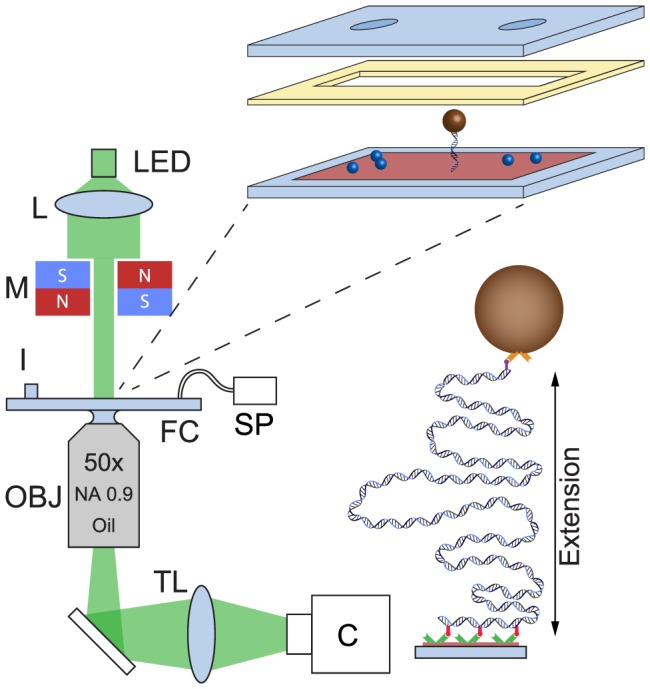
Magnetic tweezers apparatus used in this study. An LED provides illumination via a collimating aspheric lens, L, through magnet assembly M. The flow cell is imaged via a 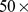 objective (Nikon NA 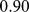 Oil) in conjunction with a 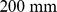 achromatic doublet tube lens, TL, onto a CMOS camera. The flow cell is constructed from two type 1 coverslips, the top one of which is sandblasted to create two 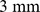 holes for fluid entry and exit. Paraffin wax film is used to separate the two coverslips and create a flow cell volume of approximately 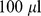. The bottom coverslip is coated with both polymer microspheres to act as reference beads and nitrocellulose. To anchor the DNA to the nitrocellulose, anti-digoxigenin is incubated in the flow cell before addition of BSA, followed later by previously built microsphere-DNA constructs. Sample is pipetted into the inlet, I, and removed via syringe pump, SP.

Magnetic tweezers have previously been described extensively [16]. Here, a multiplexed magnetic tweezers system is employed [17] with the important details described below and in figure 1. The system is based on a custom built microscope utilising a 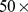 Plan NA 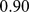 Oil (Nikon) with an achromatic doublet tube lens (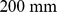) to provide 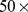 magnification. Illumination is provided from a green LED that, once collimated with an aspheric lens, is projected through the magnet assembly onto the flow cell. The magnet assembly holds two 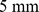 cubed NdFeB magnets (W-05-N50-G, Supermagnete, Germany) in the vertical orientation (see figure 1) [18] with vertical and angular position controlled by high-resolution translation and rotational stages (M-126.PD1, C-150.PD, Physik Instrumente, Germany). The image is focussed onto a CMOS camera (Falcon 1.4 M, Teledyne Dalsa, Germany) with images used directly for real time tracking via custom LabVIEW (National Instruments) code for immediate feedback. Compressed images are saved to disk for post processing and multiplexed microsphere tracking [17], [19].

The flow cell is constructed from two type one coverslips (BB024060S1, Fisher Scientific, Netherlands), with one sandblasted to create two 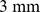 holes for flow inlet and outlet. Both coverslips are placed in an ultrasonic acetone bath for 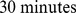 before being washed in isopropanol and left to dry. The bottom coverslip is first coated in a 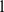 in 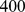 ethanol (v/v) diluted solution of polystyrene microspheres (Polysciences Europe GmbH, Germany) and heated on an 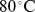 hotplate for 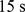, to later act as reference microspheres. Next, the same coverslip is coated in 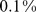 w/v nitrocellulose (LC2001, Invitrogen, USA) and heated on an 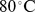 hotplate until dry. Finally, a two ply piece of paraffin wax film (Parafilm M, Bemis, USA) is sandwiched between the two coverslips and heated on an 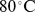 hotplate for 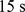 while providing gentle pressure to ensure sealing. The constructed flow cells are kept at 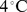 until experiments are conducted for up to two months.

### Force-extension curves

To probe and characterise the accuracy of magnetic tweezers measurements we measure and simulate force-extension curves, thus allowing us to explore a range of force-extension relations and mechanical properties.

Experimental force-extension curves of four DNA constructs, 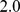, 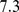, 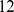 and 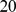 kilo base pairs (kb) in length, were measured with the following procedure. The magnet was placed at heights of 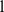 to 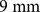 in order of increasing distance from the flow cell for a predetermined length of time (see file S1 for exact values). These magnet heights represent forces from 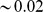 to 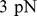 [18]. Additionally, at 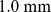 magnet height the magnets were rotated through eight full rotations at 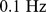 in order to fit the microsphere trajectory to a Limaçon de Pascal pattern and account for the microsphere-DNA tether attachment point [17]. The position of the probe and reference microspheres are tracked using a quadrant interpolation algorithm [19] from the previously stored images after the experiments were completed. All data was recorded at a frame rate of 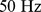, exposure time of 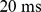 for each frame, and an acquisition time of 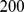 to 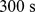.

The microsphere position data were analysed to account for camera blurring, aliasing and Faxén's correction through the method described by Velthuis et al. [16]. Only the position data for the axis parallel to the magnetic field direction is used in the calculation of forces. Following standard procedures from literature the extension of the molecule, 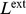, was taken as the arithmetic mean of the axial position versus time, and the force was subsequently calculated through [1], [16].
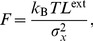
(1)where 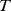 is temperature, 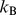 is Boltzmann's constant, and 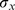 is the standard deviation of lateral position fluctuations. The WLC model can then be fitted to 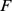 as a function of 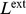 to extract 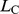 and 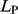 of the DNA molecule [9].

### Numerical simulations

**Figure 2:**
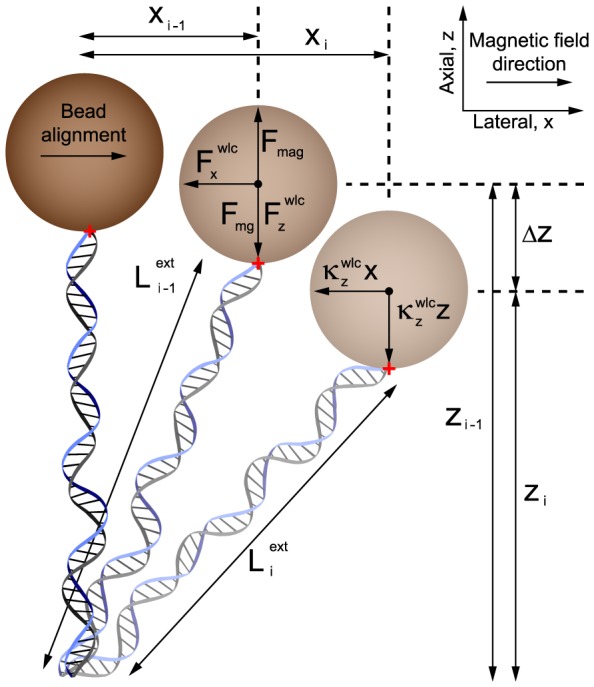
Illustration of the components considered for the time-stepping Langevin scheme used in this study. For each time step the microsphere moves to a new position due to (i) thermal noise, (ii) an elastic response from the DNA molecule that acts like an entropic spring in both 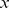 and 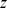, 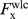 and 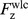, respectively, (iii) a restoring force produced from the magnetic field 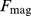, (iv) the weight of the microsphere, 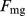, and (v) viscous drag in 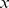 and 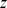, 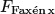 and 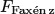, respectively. The red cross indicates the bottom of the microsphere and DNA attachment point indicating no rotation, due to the alignment of the bead in the magnetic field. Note that the microsphere here has finite extent but is treated as a point in the simulations and the y-axis has variables equivalent to those in x.

We construct a crude model of a magnetic tweezers in order to elucidate that the experimental observations are not caused by measurement errors but rather result from intrinsic biases of the method. Considering the forces that exist in the system (figure 2), the Langevin equations for the microsphere along the 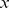
- and 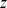-axes [20], are found to be
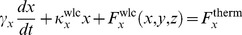
(2)and
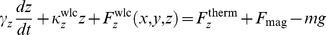
(3)respectively. Where 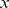 and 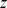 are the lateral and axial microsphere position respectively, 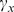 and 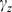 are the Faxén corrected viscous drags parallel and perpendicular to the flow cell surface, respectively (see file S1 for exact expressions), 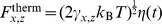, is a random Gaussian process representing thermal force noise at temperature 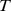 [21], 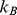 is Boltzmann's constant, 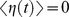 and 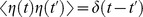, 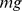 is the microsphere weight, 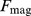 is the magnetic force, 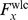 and 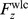 are forces arising from the entropic spring nature of the DNA in the 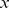 and 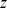 directions respectively, and 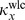 and 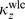 are the 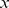 and 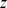 components of the WLC stiffness. This stiffness results in a restoring force upon thermal fluctuations away from the equilibrium position of the microsphere. The microsphere is treated as a point with the appropriate hydrodynamic friction such that no rotation is considered, only translation in x, y, z. Finally, a constraint is placed upon the system to exclude the volume below the coverslip as possible locations for the microsphere by repeating the previous iteration if the microsphere is in such an excluded position.

Equations 2 and 3 are solved through a finite-difference time-stepping algorithm such that the 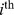 step is given by [20]

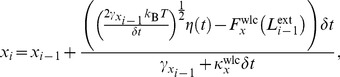
(4)with an identical equation for 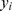 except 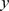 replaces all instances of 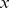, and
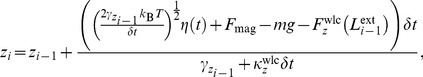
(5)where
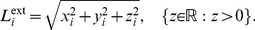
(6)


The simulations were performed in MATLAB (R2010b, The Mathworks Inc. USA) with initial parameters as follows. Total simulation time is 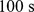, simulation time step, 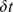, is 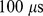, temperature, 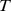, is 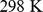, time step, 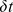, are averaged over 200 steps to create a frame rate of image acquisition is 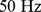 and exposure time for each frame of 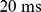, bead radius, 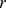, is 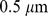 and weight is 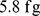. The contour length, 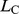, is set to that required and 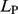 is set at 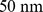. The initial axial position of the microsphere is set to that expected from the WLC model for the desired force and the initial lateral position is set to zero. The forces to simulate a force-extension curve ranged from 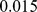 to 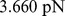, and we examined molecules of ten lengths between 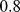 and 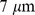 (see file S1 for exact values). The resulting data for lateral and axial position versus time was analysed in exactly the same manner as the experimental data.

### Analysis of axial position fluctuation distributions

Axial position fluctuations in magnetic tweezers have previously been assumed to describe a normal distribution given by
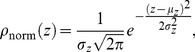
(7)where 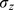 is the standard deviation and 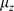 is the mean of the distribution. Via the central limit theorem one adopts the arithmetic mean, 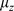, of the axial position data to represent the position of the microsphere, hence 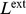. However, in this work it is shown that both experimental and simulated axial position measurements are non-normal distributions that are better described by a skew-normal distribution (from herein referred to as the skew distribution) given by,
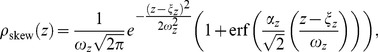
(8)where 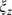 is the distribution location, 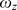 is a scale factor describing the distribution width, 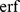 is the error function and 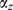 is a shape factor related to the skewness. With this distribution we take 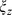 to represent the microsphere position, as it would be in the absence of Brownian motion due to a balance of opposing forces, instead of the usual 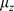. The skewness, 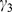, of the distribution is given as 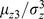, the ratio of the third moment about the mean to the standard deviation cubed, and is related to 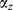, through
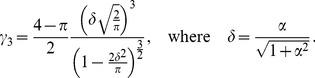
(9)


## Results

### Bias in DNA extension measurements

**Figure 3:**
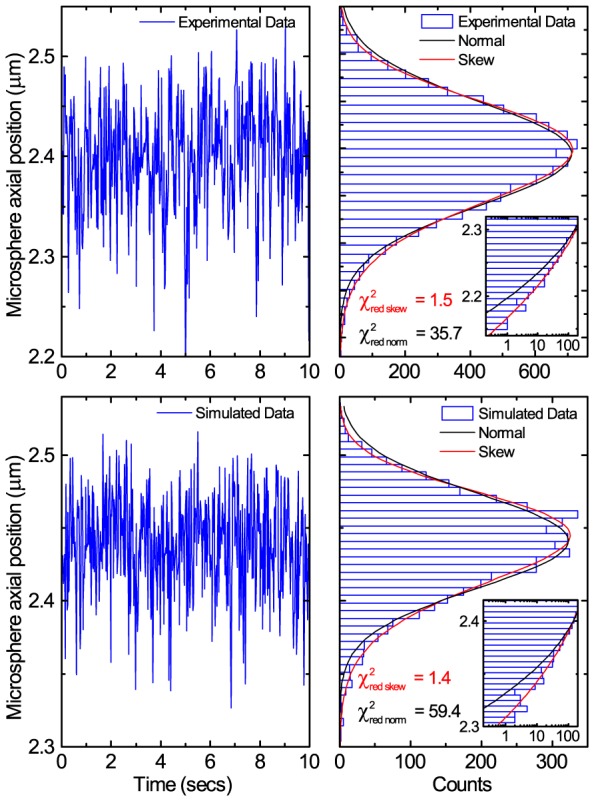
Representative examples of experimental and simulated bead height fluctuations. Top row: Representative example of experimental bead height fluctuations for the 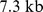 construct at a measured force of 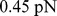. The time trace on the left is a 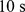 sample of the 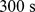 measurement. Bottom row: Simulated data for a tether with 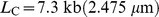 at a measured force of 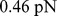. The time trace on the left is a 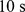 sample of the 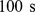 simulation. Plotting both experimental and simulated data as histograms it becomes clear from the reduced chi squared values that a skew normal distribution is a much better fit than the normal distribution to describe the microsphere axial position fluctuations. The difference between the arithmetic mean, 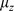, of the microsphere axial position and the skew normal distribution location, 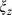, is 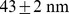 and 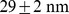 for the experimental and simulated data respectively. The inset log-linear zooms display the same data and more clearly show the large discrepancy of the data from a normal distribution, indicating that the skew normal distribution is a much better fit.

**Figure 4:**
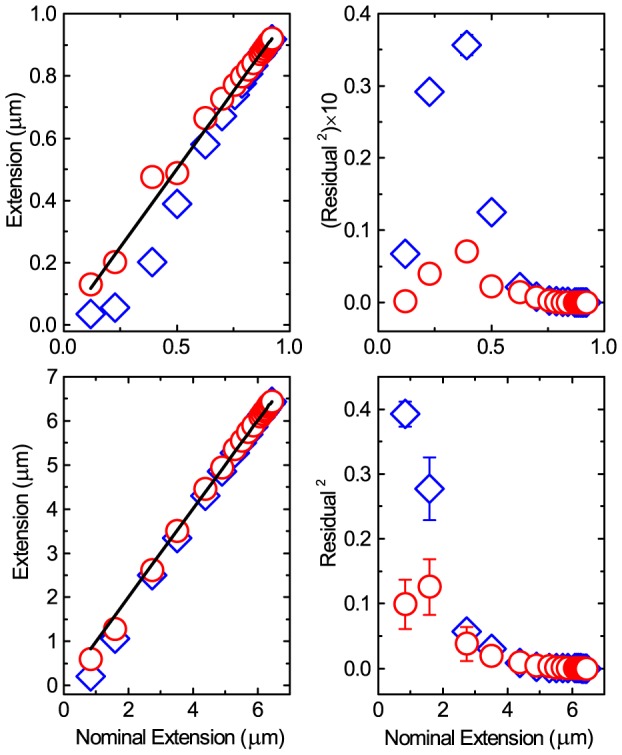
Improvement in measuring axial position using skew position shift rather than mean. Top row) Left) The calculated extension from simulations through using either the mean (blue diamonds) or the skew distribution position (red circles) as a function of nominal extension expected from the WLC, for a 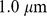 tether. The black line indicates measurement equal to the nominal extension. Right) Residuals squared for difference between measured and nominal extension using same data as left. Bottom row) Same as top except for 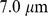 tether. Error bars are standard error of the mean with n = 5.

In figure 3 we show a representative example of microsphere axial position fluctuations versus time for an experimental, 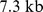 DNA construct, and a simulated DNA construct of 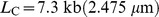 at forces of 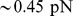. Shown to the right of each time trace are the position fluctuation histograms with both normal and skew distribution functions fitted to the data. Skewness can be observed [22]–[24] as a bias towards heights lower than the modal height in the microsphere axial position versus time traces. The effect is more clearly seen as altered tails of the position distribution histograms. For the experimental data 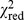 is 1.5 and 35.7 for skew and normal fits, respectively, and for simulated data 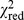 is 1.4 and 59.4, respectively. The 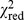 values thus clearly show the skew distribution fits the data significantly better than a normal distribution. In figure 4 the simulated DNA extension calculated through taking 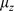 and 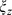 is plotted as a function of nominal extension, interpolated from the WLC [9]; i.e. the extension in the absence of Brownian motion. By taking into account skew (and correcting for external potentials, as described later) the extension measured is much closer to the nominal extension expected. This, together with the improved 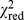 of the skew distribution, shows the location of the skew normal distribution, 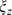, should be adopted to represent the axial position of the microsphere, rather than the arithmetic mean, 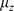. The difference between 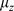 and 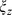 creates a systematic bias when measuring axial position and hence estimating 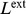. For the examples shown in figure 3 this creates discrepancies of magnitude 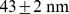 and 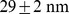 for the experimental and simulated data, respectively. Remarkably, the discrepancy in measuring 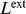 does not substantially propagate through to the calculation of applied force (figure S1). As figure S2 demonstrates, for the same physical parameters, that at short timescales noise dominates and the bias is hidden whereas at longer timescales the skewness remains.

**Figure 5:**
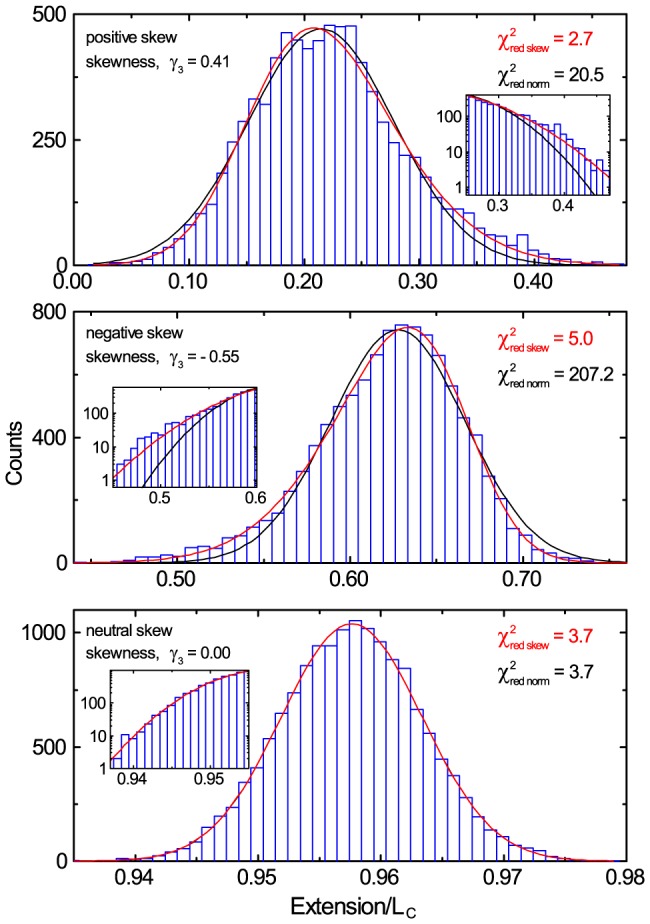
Representative experimental data for a 12 kb DNA molecule exhibiting non-normal distributions in axial position. Top) Positive skew at low force (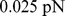) corresponds to a mismeasurement of 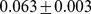 in extension/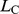. Middle) Negative skew at medium force (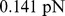) corresponds to a mismeasurement of 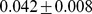. Bottom) No skew at high force (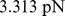). The inset log-linear zooms display the same data and more clearly show the large discrepancy of the data from a normal distribution, indicating that the skew normal distribution is a much better fit.

In figure 5 we give three examples of experimentally measured axial position fluctuation distributions demonstrating the occurrence of negative skew, positive skew, and the absence of skew. Again, the 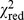 values show that a skew distribution is the better model. This further indicates that, unless realised and corrected for, the experimenter will be mismeasuring the microsphere position, thus 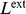, and hence mis-interpret interactions occurring.

### Skew

**Figure 6:**
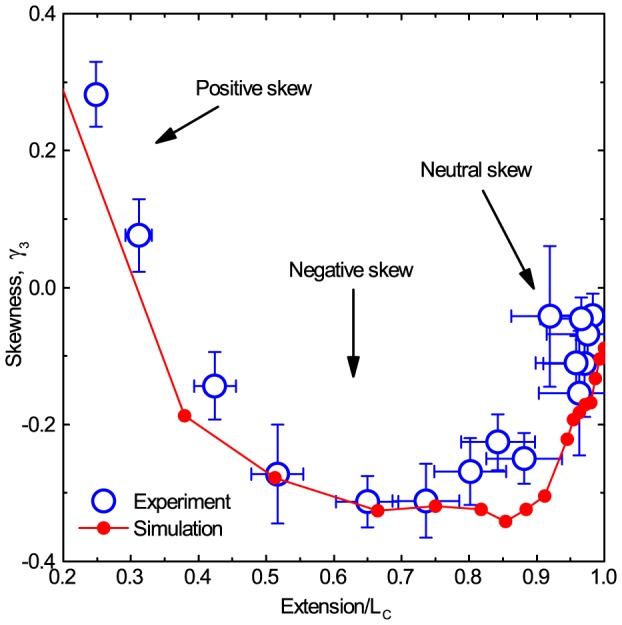
Skewness of axial position distributions as a function of DNA extension. Experimental points (blue circles) are the mean of 24 independent experiments on 12 kb DNA molecules, with standard errors of the mean displayed. Simulation data (red line) are for a molecule with 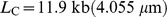, each repeated 20 times with the data analysed using the exact same method as for the experiments. Error bars are standard error of the mean.

The variation in skewness as a function of extension in figure 5 points to a more general trend that skew occurs at low forces and low extensions while the errors diminish at high forces and large extensions. To elucidate this trend further the skewness is calculated for many DNA extensions and displayed in figure 6. The data show that the magnitude and sign of the skewness of axial position fluctuations varies continuously as a function of DNA extension. There are three distinct regions. Firstly, at low extensions, or equivalently low force, the axial position distributions are positively skewed. Secondly, between 25–90% extension the distributions are negatively skewed. Thirdly, near full extension, i.e. at high force, the distributions approach zero skew and revert to normal distributions. Gratifyingly, the same trend is observed in both simulations and experiment.

### Consequences for measuring DNA mechanical properties

**Figure 7:**
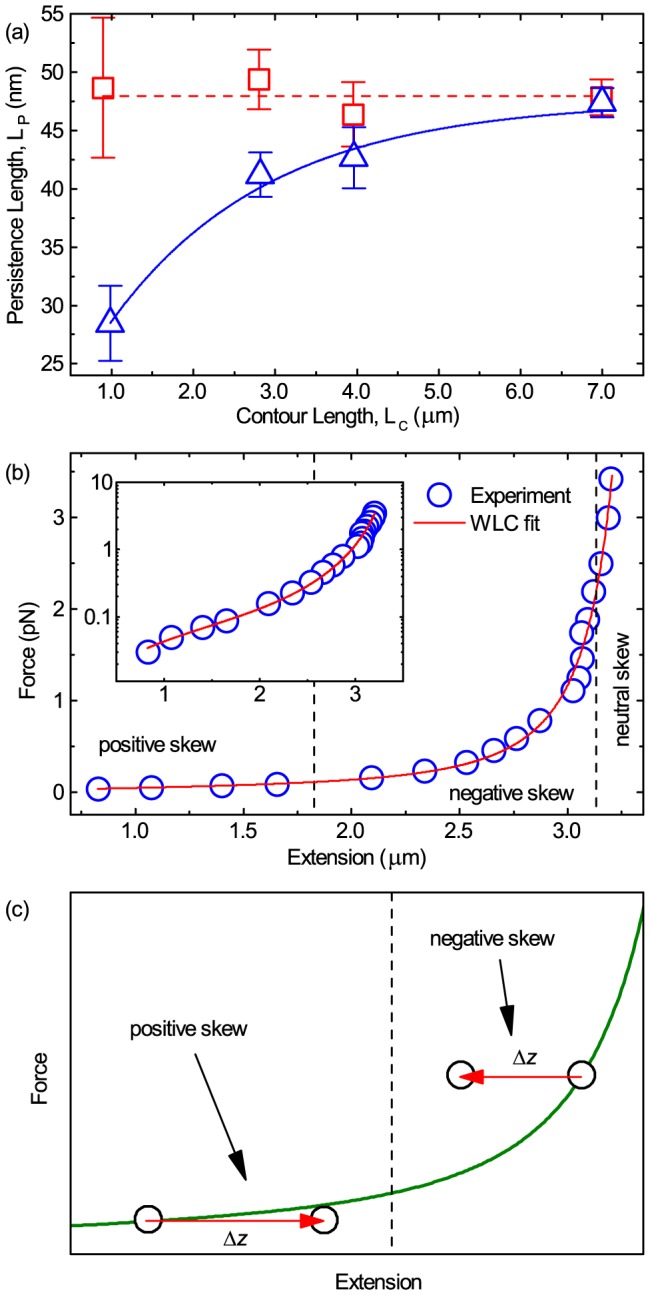
Representative fit of force versus extension data and the deduced 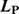. (a) Experimental uncorrected results (blue triangles) showing the variation of persistence length, 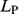, as a function of contour length, 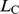, shows a pronounced and statistically significant decrease for shorter DNA constructs. Following the correction procedure in the text a corrected 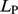 is obtained (red squares). Errors shown are standard error of the mean with 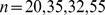 in ascending contour length. Dashed red line is 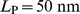, blue line is a guide to the eye. (b) Experimental force-extension curve with a WLC model fit (red line) for a 12 kb DNA molecule. For this case 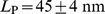 and 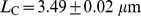. Inset) The same data on a log scale. (c) Diagram that illustrates the effect that a mismeasurement in microsphere position has upon DNA extension for positive and negative skew. The red arrows start at the extension measured using the arithmetic mean, 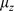, and end at the position expected if the skew normal distribution location position, 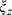, is used instead.

It is generally considered that, for constant temperature, salt concentration, and pH, that the persistence length, 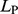, of DNA is approximately constant, with a value of 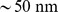 and independent of 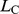, except for very short oligomers of DNA [25], [26]. Above, we have shown that significant mismeasurement in 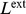 occurs through neglect of the skewed axial position fluctuations, and that this has direct consequences for the applied force deduced through equation 1. These biases, most strikingly have a pronounced effect upon the measured DNA mechanical properties. By following the standard methods of 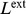 measurement, i.e. using the arithmetic mean and following equation 1, we fit the WLC model to the resulting experimental force extension curves and we discover, as can be seen in figure 7(a), that 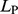 appears to vary as a function of 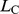.

**Figure 8:**
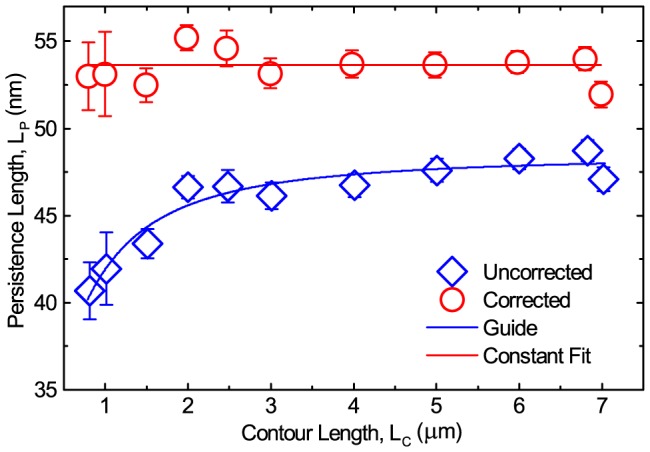
Uncorrected and corrected 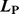 for simulated data showing the same trend as the experimental results. A decrease in measured persistence length as a function of decreasing contour length, when taking the DNA extension as the arithmetic mean 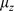 of the microsphere position data (blue diamonds). By following the correction procedure described in the text, the persistence length is corrected to a constant value for all contour lengths (red circles). Lines are guides to the eye and error bars are standard error of the mean.

Performing the same standard analysis on 20 independent simulated force versus extension data sets and plotting the simulated 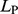 as a function of 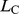 (figure 8, blue diamonds), it is again clear that erroneously low values for 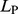 are found for low 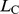 values, very similar to what is observed experimentally (figure 7(a)). Figure S3 shows typical examples of the simulated force extension data and the subsequent WLC fit for molecules of length 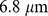 and 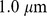. Additionally, the simulated magnetic tweezers data uses an WLC model not a finite WLC [27] so if the traditional analysis would be correct then the input parameters should be recovered, namely 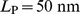. Note furthermore that previous simple simulations that neglected pendulum motion indicate the same phenomena [17] leading us to believe our explanation in the following paragraphs to be the origin of the phenomenon.

In fact by using standard calculations of force extension relationships and the subsequent WLC fits, the 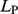 is found to decrease rapidly with decreasing 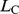, approaching about half of the expected 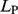, with statistical significance [28], [29], in both experiments and simulations (figures 7(a) and 8).

In order to understand the physics behind the phenomenon of these strongly deviating persistence lengths, it is informative to consider a force-extension curve in some detail. In figure 7(b) we plot a typical example of a force-extension curve for a 12 kb DNA molecule measured in the magnetic tweezers and fit with the WLC model. For this particular molecule, the characteristic properties were measured to be 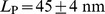 and 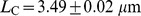. Figure 7(c) is a diagram that illustrates the change for an individual data point due to re-calculation from fitting a skew distribution and taking 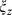, as opposed to taking 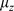. Depending on the skewness sign the extension will become either longer or shorter and hence the deduced force will increase or decrease, respectively. As a result of the adjustments in the position of the data point, the parameters of the non-linear WLC fit change, hence yielding a significantly different determination of DNA mechanical properties, 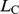 and 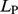.

Why are three distinct regions (figure 5 and 6) of skew observed? Firstly, looking closely at figure 7(b) it is apparent that as the extension approaches 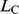 the force-extension is approximately linear. For a given extension in this region the restoring force back to equilibrium extension after a fluctuation away is thus constant for both negative and positive excursions from equilibrium. Equivalently the stiffness, or spring constant, of the entropic spring like DNA molecule is approximately constant in this region. As all other sources of force in the axial Langevin equation are either constant or stochastic and isotropic, there are no physical processes to bias the position fluctuations in one direction, and hence the distribution will not be skewed.

At low extensions in the force-extension curve, where positive skew is observed, the gradient is also approximately linear and so the stiffness is again constant. However, in this region there are two sources of anisotropic forces within the Langevin equation. Firstly, the microsphere is excluded from entering into the coverslip and so, obviously, has a bias to fluctuate in the positive direction. Secondly, the increase in hydrodynamic coupling between surface and microsphere as the microsphere approaches the surface, described by Faxén's correction [30], creates a pseudo-force in the positive direction. These two phenomena combine to produce a positively skewed normal distribution of axial position fluctuations.

Finally, at intermediate extensions, we observe appreciable negative skew. From figure 7(b), it is clear the WLC force-extension curve is non-linear in this region. Consider a microsphere under constant force in the magnetic tweezers, thus at equilibrium in extension, 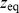, where the molecule has stiffness 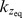. Under both positive and negative position fluctuations (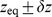) due to Brownian motion, the microsphere will experience a restoring force back to equilibrium. Specifically, under a positive position fluctuation, 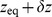, the microsphere will lie at a point on the curve that has an increased gradient in comparison to the equilibrium position, and the restoring force is from a region of higher stiffness, 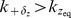. Conversely, if the microsphere undergoes a negative position fluctuation, 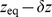, the gradient will be decreased and the microsphere experiences a restoring force from a lower stiffness region, 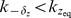. As 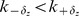 the restoring force experienced is larger for positive rather than negative excursions due to the same fluctuation, 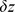. This anisotropy in restoring force gives rise to a bias towards lower extensions and hence a negatively skewed normal distribution. It is thus the non-linear DNA tether stiffness as a function of extension that underlies the phenomenon of negative skew.

### Method to reduce bias occurring from skewness

We now demonstrate a simple method to correct for the axial position mismeasurement and hence the bias in 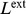, force and non-constant 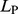. First, Faxén's correction to the perpendicular drag, 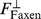, (defined fully in the file S1) is treated as a pseudo-force such that an external interaction potential, 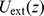, can be found through 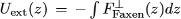. Hence a probability distribution function for the external interaction of 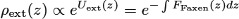 [31]. An example probability density function, 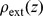, is shown in figure S4. The absolute value of this function is not needed because ultimately only 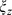 is required.

The measured probability density, or histogram, of the particle position, 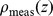, is a combination of the tether, 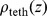, and external interaction, 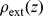, such that 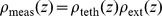. By dividing 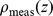 by 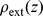 we can find the histogram that represents 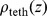 [31]. Finally, the skew-normal distribution is fit to 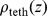 and the peak position, 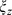, used as the DNA extension, 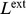, to give a more accurate representation of the expected extension (figure 4). This corrected 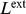 must also be used to calculate the applied force (equation 1) before fitting the WLC to the corrected data and obtaining a corrected 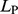 measurement (figures 7(a) and 8, red circles). Indeed we then see that the 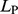 is constant as a function of 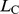. This method performs well for the experimental data and satisfactorily for the simulated data. We believe the discrepancy between the corrected simulated results and the experimental observations is due to the crude model we use. However, as we set out to qualitatively elucidate a trend as a check on the experimental observations we are gratified that the simulations match the trend of the experimental data.

## Conclusion

It is widely assumed that microsphere axial fluctuations in magnetic tweezers are normal in distribution such that the central limit theorem applies and the arithmetic mean represents the microsphere position. However, this study has shown that microsphere axial fluctuations in magnetic tweezers are non-normal in distribution. Consequently, the arithmetic mean is an inappropriate choice which leads to mismeasurement of microsphere axial position (figures 3 and 5), DNA extension, and hence forces (equation 1) and DNA mechanical properties (figures 7 and 8). It has been demonstrated that the phenomenon appears in both experiments and in numerical simulations and that the consequences can be severe, as demonstrated by a contour length dependent persistence length which can deviate by up to 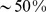 from the true value. Finally, it is demonstrated that rather than using the arithmetic mean, the location of a skew normal distribution better represents the microsphere position and hence DNA extension. Implementing this idea shows that the error can be largely resolved and recovers a contour length independent persistence length. Should the experimenter wish to accurately measure DNA extension at forces 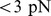, then always fit a skew normal distribution to the Faxén corrected position fluctuations and use the distribution location as microsphere position and hence DNA extension. Likewise, should the mechanical properties be extracted then a worm-like chain fit to force extension data for forces below 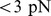 should be corrected in the manner described in this work.

## Supporting Information

Figure S1 (EPS)

Figure S2 (EPS)

Figure S3 (EPS)

Figure S4 (EPS)

File S1 Contains additional text.
(PDF)

Fig Data S1 (ZIP)
