# Supplementary material for: Skewed Brownian Fluctuations in Single-Molecule Magnetic Tweezers
Source: PLoS One. 2014 Sep 29;9(9):e108271. doi: 10.1371/journal.pone.0108271 (PMC4180755; doi:10.1371/journal.pone.0108271)
Supplement: File S1 — Contains additional text. (PDF) [file pone.0108271.s005.pdf]

Skewed Brownian fluctuations in single-molecule magnetic tweezers

Daniel R Burnham[1], Iwijn De Vlaminck[1], Thomas Henighan[1], Cees Dekker[1,*]

1 Delft University of Technology, Kavli Institute of Nanoscience, Department of Bionanoscience, Delft, The Netherlands

∗ E-mail: Corresponding c.dekker@tudelft.nl

Supporting Information

Force extension experimental measurement details

For magnet heights of 1.0 to 2.6 mm in 0.2 mm increments, 3.2 and 3.6 mm data was collected for 300 s. For 4.0, 4.4, 5.0, 5.6, 6.4 and 7.0 mm data was collected for 210 s and for 8.0 and 9.0 mm for164 s.

Force extension simulation details

The forces to simulate a force-extension curve were 0.015, 0.030, 0.059, 0.089, 0.154, 0.233, 0.353, 0.464, 0.611, 0.805, 1.217, 1.396, 1.602, 1.839, 2.110, 2.422, 2.779, 3.189 and 3.660 pN, examined for molecules of lengths 0.8, 1, 2, 2.475, 3, 4, 5, 6, 6.828, and 7 μm.

Faxén corrections

Faxén corrected viscous drag parallel and perpendicular to the flow cell surface is given by [?]

$$\gamma_{x,y} = 6\pi\rho\nu r F^{\|}_{\text{Faxen}} = \frac{6\pi\rho\nu r}{1 - \frac{9}{16}\frac{r}{z_o} + \frac{1}{8}\left(\frac{r}{z_o}\right)^3 - \frac{45}{256}\left(\frac{r}{z_o}\right)^4 - \frac{1}{16}\left(\frac{r}{z_o}\right)^5} \tag{1}$$

and

$$\gamma_z = 6\pi\rho\nu r F^{\perp}_{\text{Faxen}} = \frac{6\pi\rho\nu r}{1 - \frac{9}{8}\frac{r}{z_o} + \frac{1}{2}\left(\frac{r}{z_o}\right)^3 - \frac{57}{100}\left(\frac{r}{z_o}\right)^4 + \frac{1}{5}\left(\frac{r}{z_o}\right)^5 + \frac{7}{200}\left(\frac{r}{z_o}\right)^{11} - \frac{1}{25}\left(\frac{r}{z_o}\right)^{12}}, \tag{2}$$

where ρ is the density of the suspending medium, ν is the medium kinematic viscosity, and z_o is the distance between the microsphere centre and the flow cell surface, i.e. the arithmetic mean of axial position fluctuations or the location of the skew normal distribution.

DNA Constructs

Four DNA constructs with lengths 2.0, 7.3, 12 and 20 kilo base pairs (kb) were used in this investigation each with a single biotin on a single strand at one end of the construct and multiple digoxigenins on both strands at the opposite end of the construct.

20 kb DNA construct

A 20 kb construct was created using a PCR on Lambda pos DNA, 26531−46623 with forward primer CGT-GCGAACTCTAGATGAATTTCTGAAAGAGTTACCCCTCTAAGTAATGAGG and reverse primer BIO-TCTGGAATTGGGCAGAAGAAAACTGTCGATGCAGCCAAAATTTGTGGCGG, to create a 20093 bp fragment with a single biotin label. Additional handles were made using PCR on pBluescriptII SK+ (Stratagene) with forward primer GACCGAGATAGGGTTGAGTG and reverse primer CAGGGTCG-GAACAGGAGAGC. The PCR reaction contains 0.2 mM dNTPs (Promega) and 0.04 mM digoxigenin-11-dUTP (Roche Diagnostics). This handle (random distribution between 616 bp or 622 bp), with approximately one out of every five thymine nucleotides replaced by a digoxigenin-11-dUTP and the 20093 bp fragment are then digested using XbaI and ligated to form the construct.

12 kb DNA construct

A 12 kb construct was created using a PCR on Lambda pos DNA, 21573 − 33513 (11926 bp fragment) with forward primer BIO-CTCATGCTCACAGTCTGAGCGGTTCAACAGG and reverse primer AACGCTTCACTCGAGGCGTTTTTCGTTATGTATAAATAAGGAGCACACC, to create a 11940 bp fragment with a single biotin label. Additional handles were made using PCR on pBluescriptII SK+ (Stratagene) with forward primer GACCGAGATAGGGTTGAGTG and reverse primer CAGGGTCG-GAACAGGAGAGC. The PCR reaction contains 0.2 mM dNTPs (Promega) and 0.04 mM digoxigenin-11-dUTP (Roche Diagnostics). This handle (random distribution between 553 bp or 685 bp), with approximately one out of every five thymine nucleotides replaced by a digoxigenin-11-dUTP and the 11940 bp fragment are then digested using XhoI and ligated to form the construct.

7.3 kb DNA construct

A 7.3 kb construct was created using a PCR on Lambda pos DNA, 2808 – 10136 (7279 bp fragment) with forward primer AACTCAGCTCACCGTCGAACA and reverse primer BIO-GACGCAGGGGACCTGCAG, to create a 7329 bp fragment with a single biotin label. Additional handles were made using PCR on pBluescriptII SK+ (Stratagene) with forward primer GACCGAGATAGGGTTGAGTG and reverse primer CAGGGTCGGAACAGGAGAGC. The PCR reaction contains 0.2 mM dNTPs (Promega) and 0.04 mM digoxigenin-11-dUTP (Roche Diagnostics). This handle (random distribution between 544 bp or 694 bp), with approximately one out of every five thymine nucleotides replaced by a digoxigenin-11-dUTP and the 7329 bp fragment are then digested using pspMOI and ligated to form the construct.

2.0 kb DNA construct

A 2.0 kb construct was created using a PCR on pBluescript lambda 2,3 DNA, Lambda pos: 2808 – 4745 with forward primer BIO-GACGCAGGGGACCTGCAG and reverse primer TGTAATACGACTCAC-TATAGGG, to create a 1943 bp fragment with a single biotin label. Additional handles were made using PCR on pBluescriptII SK+ (Stratagene) with forward primer GACCGAGATAGGGTTGAGTG and reverse primer CAGGGTCGGAACAGGAGAGC. The PCR reaction contains 0.2 mM dNTPs (Promega) and 0.04 mM digoxigenin-11-dUTP (Roche Diagnostics). This handle (random distribution between 553 bp or 685 bp), with approximately one out of every five thymine nucleotides replaced by a digoxigenin-11-dUTP and the 1943 bp fragment are then digested using Xhol and ligated to form the construct.

pBluescript lambda 2,3 was constructed from pBluescriptII SK+ (Stratagene) by inserting two PCR fragments derived from λ-DNA into the multiple cloning site of the vector. PCR fragment 1 (forward primer; AAAAGAATTCGGTGACCCTTACGCGAATCC, reverse primer; AAAATCTAGAGGCTTCAGC-GACCTTGTCC) copies the 9023-11220 bp region from λ-DNA and is digested with EcoRI and Xbal. PCR fragment 2 (forward primer; AAAAGAATTCCTCAGCGACGCAGGGGACCTGCAGG, reverse primer; AAAACTCGAGTGCCGTTGTAACCGGTCATC) copies the 2804-2826 bp region from λ-DNA and is digested with EcoRI and Xhol. Digested PCR fragments 1 and 2 were cloned in the multiple cloning site of pBluescript II SK+ to create pBluescript lambda 2,3 DNA.

Tethering of DNA-microsphere constructs

Approximately 100 μl of anti-digoxigenin (Fab fragments 11214667001, Roche Diagnostics, Netherlands) (100 μg ml^{-1}) is pipetted into a flow cell via capillary action and left at room temperature for 30 minutes. Next the anti-digoxigenin is replaced with 1 ml of $T_{20}E_5$ ((20 mM) Tris pH 7.5 (Promega, H5123), 5 mM EDTA (Sigma-Aldrich, E7889)) before being replaced by bovine serum albuemin, BSA (100 mg ml^{-1}) (B9001S, Bioke, Netherlands) for 1 hour before finally being replaced by $T_{20}E_5$ and mounted in the apparatus.

To bind DNA constructs to magnetic microspheres 5 μl of 1 μm diameter magnetic microspheres (Dynabeads MyOne Streptavidin C1, Life Technologies) are washed in 100 μl of $T_{20}E_5$ + $\text{Tween}_{0.05}$ (0.05% v/v Tween 20 (Promega, H5151)) before being aggregated with a magnet in order to remove the supernatant. This is repeated three times before re-suspension in 10 μl $T_{20}E_5$ + $\text{Tween}_{0.05}$. 1 μl of DNA stock solution is added to the washed microspheres at a concentration such that the molar ratio of microspheres and DNA is approximately 1 : 1. The volume of DNA added is adjusted in order to optimise the number and quality of tethers in the field of view. The DNA and microspheres are left at room temperature for 20 minutes. The constructs are washed a final time with 59 μl of $T_{20}E_5$ + $\text{Tween}_{0.05}$ and 1 μl BSA, aggregated using a magnet and supernatant removed. Finally the constructs are re-suspended in approximately 200 μl of $T_{20}E_5$ + $\text{Tween}_{0.05}$. This final volume is also adjusted in order to optimise the number and quality of tethers in the field of view.

Tethers are created by flowing approximately 100 μl of the microsphere-DNA construct suspension into the flow cell before stopping all flow from the syringe pump and leaving the microsphere-DNA constructs for 10 minutes. Finally $\geq$ 1 ml of $T_{20}E_5$ is flowed through the flow cell to remove any non-tethered microspheres and DNA.

Figure 1. Discrepancy in measuring L_{ext} does not effect the calculated applied force. Top row) Left) The calculated force from simulations when using either the mean (blue diamonds) or the skew distribution position (red circles) as a function of simulated force input, for a 1.0 μm tether. The black line indicates measurement equal to the force input. Right) Residuals squared for difference between measured and input forces using same data as left. Bottom row) Same as top but now for a 7.0 μm tether. Error bars are standard error of the mean with n = 5.

Figure 2. Representative examples of experimental and simulated bead height fluctuations for short and long timescales. Simulated data plotted as histograms for a tether with $L_{\mathrm{C}} = 7.3\,\mathrm{kb}\,(2.475\,\mu\mathrm{m})$ at a measured force of 0.46 pN. Top) 1 s simulation, demonstrates that for short timescales noise dominates and the bias is hidden. Bottom) 1000 s simulation, which demonstrates that for long timescales the skewness is apparent.

Figure 3. Simulated force extension data and WLC fits. Typical examples of the simulated force extension data and the subsequent WLC fit for molecules of length 6.8 μm (top) and 1.0 μm (bottom). Inset) Same data on log scale.

Figure 4. Probability density function of the external interaction due to Faxén's correction. The function $\rho_{\mathrm{ext}}(z) \propto e^{-\int F_{\mathrm{Faxen}}(z)dz}$ is calculated using numerical integration.
